# The Induced Expression of *BPV E4* Gene in Equine Adult Dermal Fibroblast Cells as a Potential Model of Skin Sarcoid-like Neoplasia

**DOI:** 10.3390/ijms23041970

**Published:** 2022-02-10

**Authors:** Przemysław Podstawski, Marcin Samiec, Maria Skrzyszowska, Tomasz Szmatoła, Ewelina Semik-Gurgul, Katarzyna Ropka-Molik

**Affiliations:** 1 Department of Animal Molecular Biology, National Research Institute of Animal Production, Krakowska 1 Street, 32-083 Balice, Poland; tomasz.szmatola@iz.edu.pl (T.S.); ewelina.semik@iz.edu.pl (E.S.-G.); 2 Department of Animal Reproduction, Anatomy and Genomics, University of Agriculture in Kraków, Mickiewicza 24/28, 30-059 Kraków, Poland; 3 Department of Reproductive Biotechnology and Cryoconservation, National Research Institute of Animal Production, Krakowska 1 Street, 32-083 Balice, Poland; marcin.samiec@iz.edu.pl (M.S.); maria.skrzyszowska@iz.edu.pl (M.S.); 4 Center for Experimental and Innovative Medicine, University of Agriculture in Krakow, Rędzina 1c Street, 30-248 Kraków, Poland

**Keywords:** equine, dermal fibroblast cell, sarcoid, nucleofection, oncogenic/neoplastic transformation, RNA-Seq, NGS, transcriptome

## Abstract

The equine sarcoid is one of the most common neoplasias in the *Equidae* family. Despite the association of this tumor with the presence of bovine papillomavirus (BPV), the molecular mechanism of this lesion has not been fully understood. The transgenization of equine adult cutaneous fibroblast cells (ACFCs) was accomplished by nucleofection, followed by detection of molecular modifications using high-throughput NGS transcriptome sequencing. The results of the present study confirm that *BPV-E4*- and *BPV-E1^E4*-mediated nucleofection strategy significantly affected the transcriptomic alterations, leading to sarcoid-like neoplastic transformation of equine ACFCs. Furthermore, the results of the current investigation might contribute to the creation of in vitro biomedical models suitable for estimating the fates of molecular dedifferentiability and the epigenomic reprogrammability of *BPV-E4* and *BPV-E4^E1* transgenic equine ACFC-derived sarcoid-like cell nuclei in equine somatic cell-cloned embryos. Additionally, these in vitro models seem to be reliable for thoroughly recognizing molecular mechanisms that underlie not only oncogenic alterations in transcriptomic signatures, but also the etiopathogenesis of epidermal and dermal sarcoid-dependent neoplastic transformations in horses and other equids. For those reasons, the aforementioned transgenic models might be useful for devising clinical treatments in horses afflicted with sarcoid-related neoplasia of cutaneous and subcutaneous tissues.

## 1. Introduction

Sarcoid is one of the most common skin tumor types in equids. It does not belong to the metastasizing tumors but is considered to be locally invasive [1,2,3]. Moreover, the high severity rates that have been found to result from sarcoid-dependent oncogenic transformation of epidermal and dermal tissues seem to be low. However, sarcoids contribute to lowering the value of the animal and the overall deterioration of the animal’s welfare by occurring mainly in places exposed to movement [2]. This location exposes the possibility of mechanical damage, which can lead to transformations of minor forms into severe forms characterized by ulceration [2].

So far, it has been possible to link the presence of sarcoids with the infection of bovine papillomavirus types 1 and 2 (BPV-1, BPV-2) and, less frequently, type 13 (BPV-13), which has been confirmed at the DNA, miRNA, and protein levels [3,4,5]. These viruses belong to a species-specific family of *Papillomaviridae*, which means they can only infect specific species of animals. BPV is so far the only documented case of a natural species barrier breach [6]. In cattle, it typically attacks the differentiated cells stemming from epithelial and connective tissues such as epidermal keratinocytes and dermal fibroblasts [6,7], causing mainly skin lesions in the form of papillomas, warts, and various neoplasms [8]. In the family *Equidae*, the infection does not produce new virus particles [9], but leads to changes in the level of gene expression, leading to neoplastic changes.

The genome of *Papillomaviridae* is highly conserved. It consists of seven early genes (*E1–E7*), two late genes (*L1* and *L2*), and a noncoding region (NCR, also known as the upstream regulatory region or URR) containing regions that control viral replication. The early genes are responsible for the replication activities of the virus (*E1*), the regulation of transcription (*E2*), and the coding of individual viral proteins (cytoplasmic—*E3*, and transforming—*E4*–*E7*). Late genes encode viral capsid proteins [10,11]. Among the transforming proteins, the *E5–E7* proteins are found in the genomes of all known carcinogenic viruses [12].

There are many known treatment methods for dealing with sarcoids. Unfortunately, some of them are only effective for a specific type of sarcoid and only for a specific tumor site (such as the BCG vaccine) [13]. Surgical methods have a high probability (up to 30%) of the disease’s recurrence in a more aggressive form [14]. There are also methods with good prognosis, but due to the need to apply them directly to the skin lesion, they can be very painful; also some sites, like ears, are more sensitive, which requires general anesthesia in certain cases [15]. For this reason, further efforts are needed to develop new treatments for this condition, which could be amended by the development of new models that can study that neoplasm at the molecular level.

The molecular mechanisms underlying sarcoid-dependent neoplastic transformation are not yet fully understood. Previous studies have focused on the analysis of differences in gene expression between sarcoid and normal skin tissues, comparing the transcriptional activities of genes in the cell lines established from these tissues [16,17]. Some studies have aimed to devise in vitro models of murine fibroblast-derived cancerous cell lines generated by transfection with *BPV* transgenes or to create mouse models of dermal sarcoid-related neoplasia [18]. However, so far there have been no studies that target the development of an ex vivo model of sarcoid-dependent tumorigenesis in equine adult cutaneous fibroblasts cell (ACFC) lines, whose oncogenic transformation has been accomplished by transfection with *BPV* fusion genes. Moreover, there has been a lack of data confirming which virus genes are responsible for the neoplastic transformation of ACFCs into dermal sarcoid-like cells. That model could contribute to a more comprehensive understanding of the molecular changes in equine ACFCs undergoing sarcoid-related cancerogenesis due to viral infection.

Multifaceted transcriptomic characterization of mitotically stable cancerous cell lines stemming from *BPV-E4* and *BPV-E4^E1* transgenic equine ACFCs that have undergone nucleofection-mediated neoplastic transformation into nonmalignant sarcoid-like tumor cells is a sine qua non for accomplishing somatic cell cloning. The use of ACFC-derived sarcoid-like cells as a completely new source of nuclear donor cells (NDCs) to create equine cloned embryos and progeny by somatic cell nuclear transfer (SCNT) has not yet been realized. On the other hand, efforts by Li et al. [19] and Shao et al. [20] have confirmed that successful transcriptional reprogramming and molecular dedifferentiation of genomes inherited from NDCs that originated, respectively, from such highly metastatic neoplasms as cerebellum-specific medulloblastoma and breast cancer have sustainably contributed to promoting the epigenetically controlled remission of their typically cancerous markers and malignancy-related attributes in cloned mouse embryos.

To the best of our knowledge, transgenization and simultaneous co-transfection of the ex vivo expanded equine ACFCs that have been created by nucleofection according to the approaches formerly devised and adapted by Skrzyszowska et al. [21] and Samiec et al. [22] to generate genetically modified cloned pig embryos have not yet been reported. Additionally, the aforementioned strategies have been applied, for the first time, to research targeted at not only cell culture engineering but also experimental and preclinical attempts, with the use of in vitro transgenic models designed to examine the molecular nature of sarcoid-dependent oncogenic transformation (carcinogenesis) of equine ACFCs. These extracorporeal models have also been developed to explore the genetic and epigenetic determinants of procancerous tumorigenesis of epidermal and dermal provenance in horses and phylogenetically consanguineous taxa (i.e., other equids).

Furthermore, it is also noteworthy that, thus far, approaches focused on utilizing *BPV-E4* and *BPV-E4^E1* transgenic ACFC derivatives, which have undergone oncogenic transformation into sarcoid-like cells as a result of nucleofection, have been conceptualized for the needs of SCNT-based cloning in horses and a variety of members of *Equidae* family for the first time. For all these reasons, elaborating the abovementioned approaches seems to be strongly justified by the scientific thesis assuming profound amelioration of epigenomic plasticity in the ex vivo immortalized nonmalignant cancerous derivatives of ACFCs, which are characterized by an unlimited lifespan. This, in turn, might result in the enhanced susceptibility of genetically modulated ACFC-derived sarcoid-like cell nuclei to being epigenomically dedifferentiated and transcriptionally reprogrammed in equine cloned embryos generated by SCNT-mediated assisted reproductive technologies (ARTs).

Therefore, in our current investigation, efforts were undertaken to generate equine ACFC lines that had been genetically transformed into sarcoid-like cells as a result of their nucleofection with *BPV* transgenes encoding recombinant representatives of the transforming protein family, designated as either *BPV-E4* or *BPV-E1^E4*. Our study also sought to thoroughly unravel the modifications arising in genomic signatures that have incurred sarcoid-dependent alterations in transcriptomic profiles of horse ACFC-derived neoplastic cells.

## 2. Results

### 2.1. Preliminary Validation of the Samples Used

All the harvested horse skin tissues were tested for the presence of *BPV* DNA. The presence of the viral DNA amplicon in samples intended for further procedures showed the absence of products unique for *BPV* genetic material.

### 2.2. Comparative Statistical Estimations Resulting from Next-Generation Sequencing (NGS) among BPV-E4 and BPV-E1^E4 Transgenic Equine ACFC-Derived Neoplastic Cells

After NGS sequencing, an average of 13 million raw reads were obtained per sample, from which 99.6% passed the quality filters. The mapping rate to reference genome ranged from 73.6% to 89.1% (average: 86.8%), which was about 11.3 million reads mapped per sample. The PCA clustering that was performed for both comparisons confirmed the group homogeneity (Figure S1). The average percent of reads mapped to genes per sample was 65.5. The data have been submitted to the Gene Expression Omnibus (GEO) database and received the accession number GSE193906.

### 2.3. Analysis of Differentially Expressed Genes (DEGs) in Oncogenically Transformed Equine ACFCs Expressing BPV-E4 and BPV-E1^E4 Transgenes

Transcriptome profiling allowed us to perform a comparison of the whole expression profile between the control and the *BPV-E4* and *BPV-E1^E4* groups. After the comparison of control and *BPV-E4* groups, 1640 DEGs were identified, of which 624 were upregulated and 1016 downregulated in the *BPV-E4* group. The highest numbers of 3328 DEGs were identified due to the comparison of the control and *BPV-E1^E4* groups. Among them, 1626 genes were shown to be upregulated and 1602 genes were recognized to be downregulated in the *BPV-E1^E4* group as compared to the control samples.

**Figure 1:**
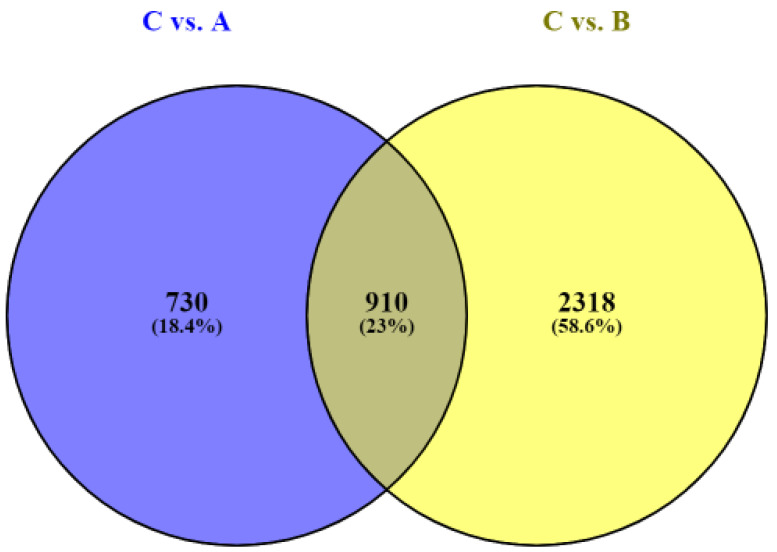
The Venn diagram of common and unique differentially expressed genes (DEGs) following the comparisons of *BPV-E4* (A) vs. control (C) groups, and *BPV-E1^E4* (B) vs. control (C) groups (Venny 2.1 BioinfoGP).

To establish the differences between the impacts of *BPV-E4* and *BPV-E1^E4* transgenes, the DEG sets obtained for both comparisons were combined and 910 common genes were identified. Moreover, 2318 and 730 unique DEGs were detected following sarcoid-dependent neoplastic transformation of equine ACFCs via nucleofection with *BPV-E1^E4* and *BPV-E4* transgenes, respectively (Figure 1).

**Figure 2:**
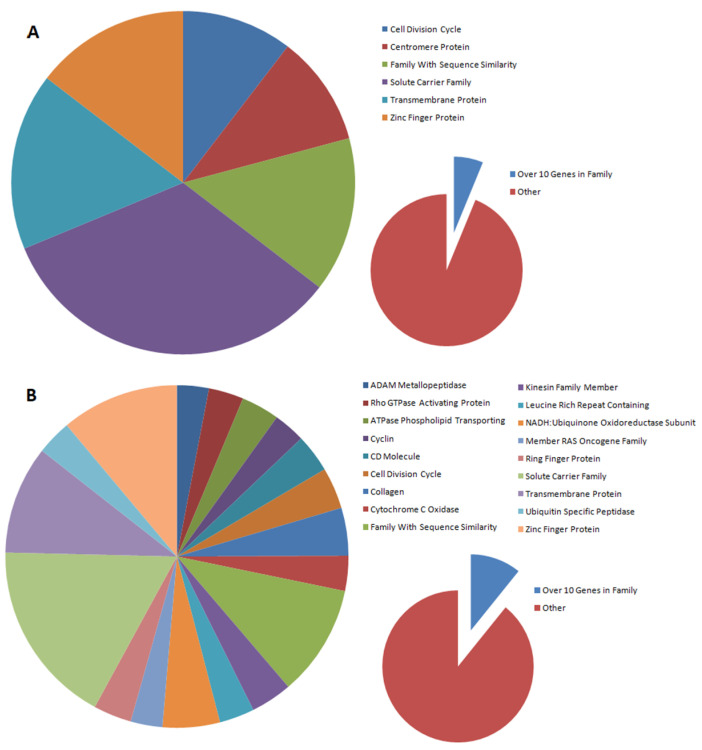
Pie charts that depict the contribution of families with 10 or more genes undergoing expression changes identified following the sarcoid-dependent oncogenic transformation of equine ACFCs triggered by nucleofection with either *BPV-E4* (**A**) or *BPV-E1^E4* (**B**) transgenes.

Among the DEGs identified for cells nucleofected with the *BPV-E4* transgene, six gene families were found, for which the expression of 10 or more genes was altered, and they accounted for 6% of all DEGs (Figure 2A). In the case of *BPV-E1^E4* transgene-mediated nucleofection, there were three times as many such families, and they accounted for 10% of all DEGs (Figure 2B). Only one family encompassing the genes encoding centromere proteins identified for *BPV-E4* transgenic samples did not occur among the gene families identified for *BPV-E1^E4* transgenic cell counterparts.

### 2.4. Gene Ontology (GO) Enrichment Analysis of BPV-E4 and BPV-E1^E4 Transgenic Equine ACFC-Derived Neoplastic Cells

The GO enrichment analysis performed for DEGs between control and *BPV-E4* groups allowed us to detect several significant Gene Ontology terms (Table 1). Most of those GO terms were over-represented as follows: 34 DEGs correlated to negative regulation of cell proliferation (FDR < 0.002); 24 DEGs responsible for positive regulation of cell migration (FDR < 0.0001); and 21 DEGs related to both cell adhesion and cell migration (FDR < 0.003 and FDR < 0.0005, respectively). Additionally, the overabundance of DEGs that has been shown to be significant was noticed for GOs characteristic of cell–matrix adhesion and actin cytoskeleton organization. In identified GO terms, the genes that represented two families have been found to occur frequently, as has been indicated below: the genes coding for different isotypes of integrins (*ITG*s) such as *ITGB6*, *ITGB3*, *ITGA6*, *ITGA1*, *ITGA8*, and *ITGB4*, and the genes coding for kinesin superfamily proteins (*KIF*s) such as *KIFC1*, *KIF23*, *KIF11*, *KIF20A*, and *KIF3B*.

The enrichment analysis performed for the comparison of the incidence of DEGs between control and *BPV-E1^E4* samples revealed that the focal adhesion GO terms were over-represented by 99 DEGs, 65 of which were upregulated; their 34 counterparts were downregulated (FDR < 0.0001) (Table 2). The GOs, for which most of the genes were recognized as upregulated, have been shown to be related to negative regulation of extrinsic apoptotic signaling, transforming growth factor-β receptor signaling, and collagen fibril organization.

### 2.5. Pathway Enrichment Analysis among Oncogenically Transformed Equine ACFCs Expressing BPV-E4 and BPV-E4^E1 Transgenes

The results confirmed almost the same significantly over-represented molecular pathways, not only between *BPV-E4* and control intergroup comparisons (Table 3), but also between *BPV-E1^E4* and control intergroup comparisons (Table 4). The PI3K-Akt signaling pathways were identified with the highest numbers of DEGs–44 for *BPV-E4* and 73 for *BPV-E4^E4* transgenic cell subpopulations, respectively. In both cases, the genes encoding integrins (*ITG*s) and fibroblast growth factors (*FGF*s) were detected.

**Figure 3:**
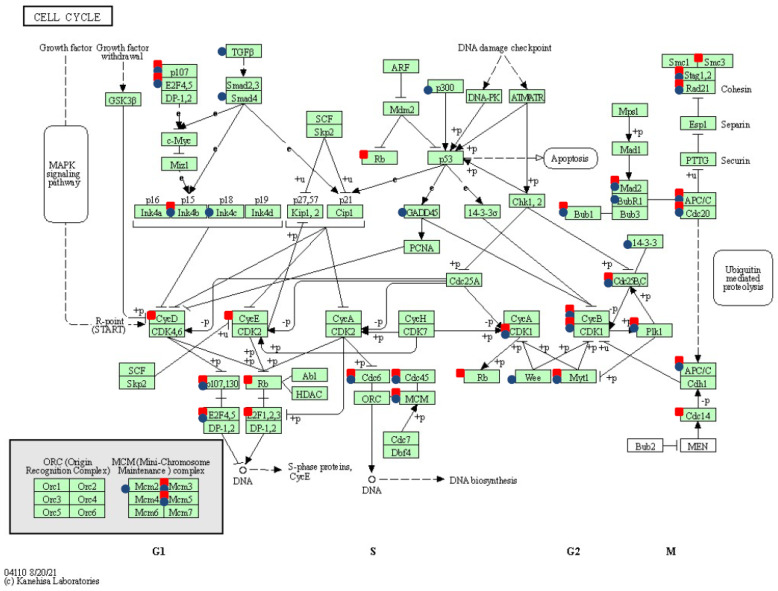
Cell cycle pathway (ecb 04110) over-represented following two strategies of sarcoid-dependent neoplastic transformation triggered by either *BPV-E4* or *BPV-E1^E4* transgenes. The red squares denote genes modified by *BPV-E4* insert, while the blue circles indicate *BPV-E1^E4* transgene-induced modifications; arrows present molecular interaction or relation, while dotted arrows show indirect link or unknown reaction.

**Figure 4:**
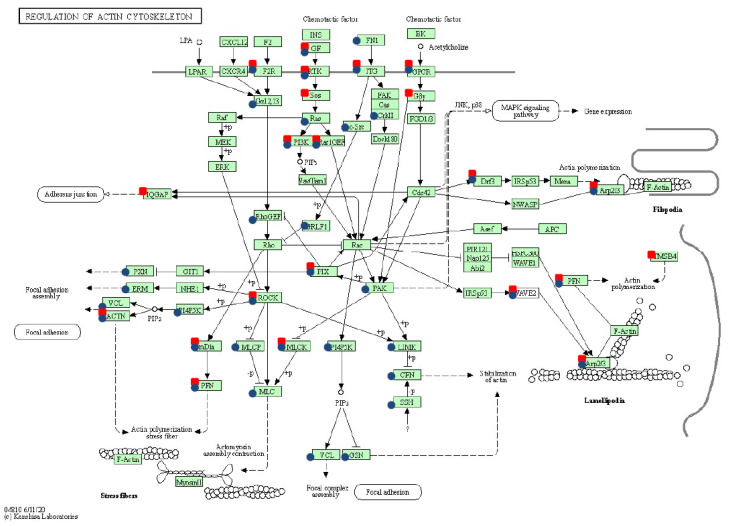
Regulation of actin cytoskeleton pathway (ecb04810) modifications following two strategies of sarcoid-dependent neoplastic transformation triggered either by *BPV-E4* or by *BPV-E1^E4* transgenes. The red squares denote genes modified by *BPV-E4* insert, while the blue circles indicate *BPV-E1^E4* transgene-induced modifications; arrows present molecular interaction or relation, while dotted arrows show indirect link or unknown reaction.

Furthermore, as has been revealed by the pathway enrichment analysis, modifications observed in the cell cycle (Figure 3), regulation of actin cytoskeleton (Figure 4), and ECM remodeling (reflected in the alterations recognized for focal adhesion and ECM-receptor interaction) have been proven among neoplastically transformed equine ACFCs exhibiting expression of either *BPV-E4* or *BPV-E1^E4* transgenes.

**Figure 5:**
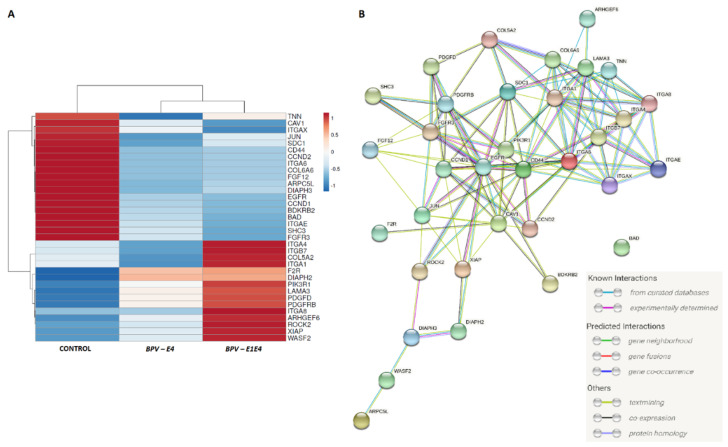
The interactions between 34 genes identified in both *BPV-E4* and *BPV-E1^E4* transgenic cells, and belonged to focal adhesion, regulation of actin cytoskeleton, and ECM-receptor interaction pathways. (**A**) the heatmap of the mean expression for groups (R package [23]); (**B**) the inter-relations between identified DEGs (String software [24]).

The DEGs associated with such processes as focal adhesion, regulation of actin cytoskeleton, and ECM-receptor interaction were represented mainly by integrins, lamins, collagens, and *FGF* genes (Table 3 and Table 4). Taking into account these pathways, for cells transformed oncogenically via nucleofection with *BPV-E4* and *BPV-E1^E4* gene constructs, the most upregulated genes detected are *ITGA8* (*Integrin Subunit Alpha 8*), *XIAP* (*X-Linked Inhibitor Of Apoptosis*), *ROCK2* (*Rho Associated Coiled-Coil Containing Protein Kinase 2*), and *LAMA3* (*Laminin Subunit α3*), while such genes as *FGF12* (*Fibroblast Growth Factor 12*), *FGFR3* (*Fibroblast Growth Factor Receptor 3*), *ITGA6* (*Integrin Subunit α6*), *CCND1* (*Cyclin D1*), *CCND2* (*Cyclin D2*); *COL6A6* (*Collagen Type VI α6 Chain*), and *BAD* (*BCL2-Associated Agonist Of Cell Death*) have been allotted to their downregulated counterparts (Figure 5A,B).

The onset of pathways related to cancerous transformation was identified uniquely for genetically transformed cells that had been nucleofected with *BPV-E4* gene construct (Table 3). The significant overabundance in initiating of pathways associated with neoplasia has been empirically justified by the detection of 52 DEGs in *BPV-E4* transgenic equine ACFCs oncogenically transformed into sarcoid-like cells (Table 3). Only *BPV-E1^E4* transgenic cells were characterized by promoting and rewiring molecular programs based on the FoxO-, Rap1-, and TNF-mediated signaling pathways and molecular mechanisms dependent on proteoglycans actively functioning in cancer cells (Table 4). The switching on of procancerous mechanisms prompted by Rap1 signaling pathway and activation of proteoglycans is reflected in the presence of 49 and 48 DEGs, respectively. Crosstalk between these molecular regulatory networks in *BPV-E1^E4* transgenic equine ACFC-derived neoplastic cells remains under control and requires the reciprocal cooperation of the panel of genes linked to Wnt signaling pathway and coding for such proteins as fibroblast growth factors, matrix metalloproteinases (*MMP*s), and interleukins (Table 4).

### 2.6. Enrichment Analysis for Identification of DEGs in BPV-E4 and BPV-E1^E4 Transgenic ACFCs Undergoing Sarcoid-Dependent Oncogenic Transformation

**Figure 6:**
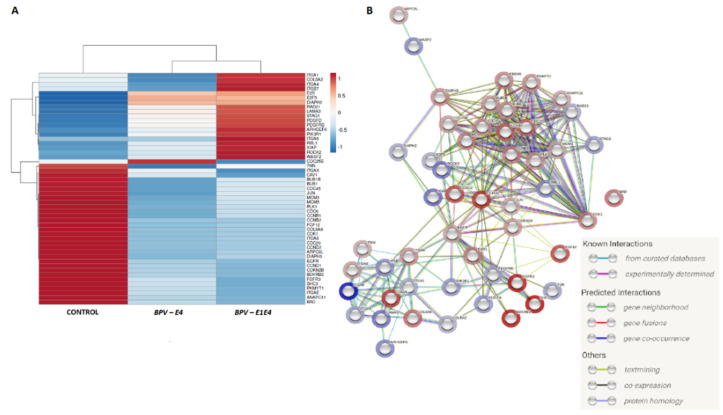
The interactions between 51 genes identified in both *BPV-E4* and *BPV-E1^E4* transgenic cells undergoing sarcoid-dependent neoplastic transformation, and belonged to molecular pathways related to procancerous intracellular conversion: (**A**) the heatmap of the mean expression for groups (R package [23]); (**B**) the interaction between identified DEGs with FC direction marked (String software [24]).

The 910 genes identified as differentially expressed in both *BPV-E4* and *BPV-E1^E4* genetically transformed groups as compared to the control were analyzed in terms of enrichment pathways and GO terms. The results confirmed the significant incidence of DEGs associated with cell cycle control (FDR < 0.0001, 21 DEGs), regulation of actin cytoskeleton (FDR < 0.0007; 21 DEGs), and focal adhesion (FDR < 0.0009; 21 DEGs). The genes involved in these molecular networks displayed close interactions and were simultaneously characterized by the occurrence of two clusters dependent on gene association and direction of modifying/diversifying their transcriptional activities (Figure 6A). The genes with the highest number of interactions with other DEGs were either downregulated as follows: *CDK1* (*Cyclin-Dependent Kinase 1*), *EGFR* (*Epidermal Growth Factor Receptor*), *CCND1* (*Cyclin D1*), and *CCND2* (*Cyclin D2*); or upregulated as follows: *ITGA8* (*Integrin Subunit Alpha 8*) and *RLB1 gene* (Figure 6B).

### 2.7. qPCR-Assisted Validation Accomplished for Transcriptional Activity Levels of Genes in Neoplastically Transformed Equine ACFCs Expressing BPV-E4 and BPV-E1^E4 Transgenes

The qPCR validation confirmed a high and significant correlation between RNA-seq data and relative quantities/abundances of gene transcripts estimated using real-time PCR methods (Table 5). The highest correlation coefficients have been identified for *TIMP1*, *MMP2*, *MMP14*, and *MMP24* genes (*R^2^* from 0.814 to 0.989). The occurrence of a nonsignificant correlation coefficient between RNA-seq- and qPCR-mediated expression profiles was noticed for only one gene, *MMP17*.

## 3. Discussion

To date, there is still limited information about equine sarcoid genetics as well as about the etiology of sarcoids’ occurrence at the molecular level. The identification of such mechanisms related with neoplasia formation seems to be critical for prophylaxis or future treatment. Little research has been done comparing the sarcoid cell transcript with that of healthy horse skin cells. These studies were mainly performed on microarrays, so they were limited by the selected panel of genes [16]. Transfected horse skin cells are proposed as a new model to conduct research on the effect of individual viral genes on changes inside the cell. In this study, we compared the overall transcriptome using the RNA-seq technique, which enabled the detection of DEGs on a much larger scale. We tried to answer the question of which viral genes affect the cell transcriptome, directing changes toward neoplastic transformations, and how. So far, a model of transfected skin cells has been developed in sarcoid research, but it included transfected mouse skin cells [18]. Moreover, this model was not conducted fully in vitro due to the introduction of altered cells into living organisms. An additional advantage of the present research was using two variants of the studied transcript. Such an approach made it possible to approximate the functions performed by the added fragment of the E1 protein. So far, it has been argued that the effect of the papillomavirus E4 protein is mainly related to viral replication by controlling cellular processes towards the return of differentiated cells to the cell cycle [25,26,27]. Our research has demonstrated that the presence of the BPV-E1^E4 protein also influences changes in the expression of other host cell genes and may play a role in carcinogenesis.

Following *BPV-E4* transgene-mediated nucleofection of equine ACFCs, a total of 1640 DEGs were identified, out of which 62% were found to be downregulated and 38% upregulated. In contrast, after *BPV-E1^E4* transgene-dependent neoplastic transformation of ACFCs into sarcoid-like cells, 3328 genes were detected, out of which 51% were shown to be downregulated and 49% upregulated. This confirms the ratio of downregulated genes to upregulated genes obtained in the microarray studies performed by Semik et al. [16] and in other cancers such as pancreatic cancer, cervical cancer, and renal cancer [28,29,30]. Attention should be paid to the differences in deregulated genes depending on the type of insert introduced. In the case of the fragment encoding the BPV-E4 protein alone, genes deregulated also by the splicing protein BPV-E1^E4 accounted for 55%, while genes common to both inserts accounted for 27% of all deregulated genes. Therefore, the conclusion is drawn that the presence of the BPV-E1 protein fragment strongly influences the change of the protein function in the process of neoplasia formation, not only by enabling modification of the expression level of new genes but also by inhibiting the deregulation of gene expression occurring in the case of the direct product of the *BPV-E4* gene.

The distribution of gene families that are differentially expressed between nontransfected and transfected cells depends on the type of gene introduced. In our studies, gene families were selected in which at least 10 genes were subject to altered expression. In the case of the introduction of the *BPV-E4* gene alone, six gene families were observed to be differentially expressed (cell division cycle, centromere protein, family with sequence similarity, solute carrier family, transmembrane protein, and zinc finger protein), while for the spliced insert, three times as many families were detected (ADAM metallopeptidase, Rho GTPase-activating protein, phospholipid-transporting ATPase, cyclin, CD molecule, cell division cycle, collagen, cytochrome c oxidase, family with sequence similarity, kinesin family member, leucine-rich repeat-containing protein, NADH:ubiquinone oxidoreductase subunit, member of RAS oncogene family, ring finger protein, solute carrier family, transmembrane protein, ubiquitin-specific peptidase, and zinc finger protein). Moreover, all families (with the exception of centromere proteins) designated for the *BPV-E4* insert were also among the families designated for the *BPV-E1^E4* insert. This may indicate that, despite the differences in DEGs, the major pathways regulated by this protein are not altered by the introduction of the BPV-E1 protein fragment to cells, but the number of such families is increasing.

Interesting results were obtained based on the Gene Ontology analysis. The function of the BP virus E4 protein is related to the reintroduction of the host cells into the cell cycle [27]. For the *BPV-E4* insert, 34 DEGs were identified for GO regulation of cell proliferation, 11 DEGs for chromosome segregation, nine DEGs for the mitotic spindle assembly, and eight DEGs for the mitotic cytokinesis that can be associated with this function. However, we cannot unequivocally determine whether the differences noticed in the transcriptomic profiles exert a negative or positive effect on the host cell cycle. Our research also showed that a similar number of statistically significant changes in GO were seen for the DEGs associated with cell migration processes such as positive regulation of cell migration (24 DEGs), cell adhesion and cell migration (21 DEGs respectively), and cell–matrix adhesion (15 DEGs). This may indicate that the reasons for the nonmetastatic nature of the sarcoid [2] can be found in the analyzed gene. Among the DEGs belonging to the changed GOs, the protein families of integrins and kinesin superfamily proteins had the largest share. The high proportion of integrins may indicate the neoplasmic nature of the BPV-E4 protein. Changes in the expression level of integrins have been associated with various cancers. They act as a factor controlling the migration capacity of altered cells via extracellular matrix (ECM) remodeling and modification of cell–ECM interaction [31,32,33]. It has been established that integrin also plays a key role in the regulation of cancer progression through involvement in the regulation of cancer stem cells, metastasis, tumor angiogenesis, and metabolism [33]. In turn, kinesin superfamily proteins are involved in transporting many intracellular components. Additionally, they are involved in cell division and are responsible, among other things, for the assembly of microtubule spindles and the separation of chromosomes. Their expression is tightly regulated and its disturbance can lead to increased (in the case of upregulation) or decreased (downregulation) cell proliferation [34].

In the case of the *BPV-E1^E4* insert, the largest number of DEGs were involved in focal adhesion, with 99 genes in total, and nearly twice as many genes were upregulated. This may indicate a high involvement of the BPV-E1^E4 fusion protein in the processes related to cells migration. Another significant GO was the negative regulation of the canonical Wnt signaling pathway. Deregulation of this pathway is associated with the formation and metastasis of numerous cancers, such as colorectal cancer, breast cancer, and ovarian cancer [35]. An example that can be drawn from our present study is the overexpression of the *SOX9* gene, which is considered a tumor progression factor [36]. The results of the research by Aldaz et al. [36] proved that an increased level of *SOX9* can promote tumor cell proliferation in both in vitro and in vivo models throughout *BMI1* activation and *p21* inhibition. The study by Xue et al. [37] pinpointed the strong influence of the overexpression of *SOX9* on breast cancer stem cells, while in the report by Ma et al. [38], *SOX9* was designated as a “master regulator” of the processes encompassing the survival and metastasis of breast cancer cells [38].

Additionally, GOs, whose deregulation is associated with carcinogenic processes, such as apoptosis, and which are classified as related to hallmarks of cancer [39], and the *TGF-β* (transforming growth factor-β) receptor (*TGFBR*) signaling pathway, have been shown to be significant. The *TGF-β* gene is considered to be one of the most potent regulators of cell proliferation (usually negative), and it can also function as a promoter of the metastasis of TGF-β-resistant tumor cells [40]. Several previous reports indicated that upregulation of the *TGFβ1* gene is characteristic during tumorigenesis and can promote cell motility and migration [41,42]. Moreover, in vitro studies by Zhou et al. [43] have confirmed that the transfection of neoplastic (adenocarcinoma) cells derived from colonic and rectal epithelial cells (enterocytes) with the use of a pCMV5-TGFBR1*6A-HA gene construct brings about TGFBR1*6A (type 1 transforming growth factor β receptor)-induced activation of the p38 MAPK pathway, followed by expedited and highly malignant oncogenic modulation of these colorectal tumor cells. This, in turn, gives rise to an enhancement of the ex vivo capabilities of colorectal cancer cells to grow unchecked, invade less invasive or noninvasive subpopulations of intestinal adenocarcinoma cells, and metastasize from primary malignant lesions (i.e., primary tumor sites) to other locations (the so-called metastatic foci) of the transgenic cell culture engineering model [43]. The upregulation of the *TGFBR* gene, which was observed in our study due to the *BPV-E1^E4* transgene-mediated oncogenic transformation of equine ACFCs into sarcoid-like cells, may also indicate a pivotal role of TGF-β receptors in the onset and progression of the processes responsible for the migration and metastasis of neoplastic cells. The other upregulated gene, which represents the GOs related to *TGF-β* receptors, is the *c-Fos* proto-oncogene, widely recognized as one of the most important predictors determining carcinoma’s progression [44]. The exact role of the *FOS* gene in tumorigenesis and metastasis is still unclear, but the overexpression of this gene has been hypothesized to trigger tumorigenesis and, thereby, has been potentially found to be a poor prognostic factor for oncology patients. The increased expression of the *FOS* gene can trigger the *VEGF* (vascular endothelial growth factor) and enhance *NANOG* and *c-myc* genes in head and neck squamous cell carcinoma [45]. On the other hand, downregulation of the *Fos* proto-oncogene can be related to tumor suppression [46].

The whole transcriptome’s modification under both transfection types showed the significant overexpression of genes involved in pathways related to cytoskeleton and ECM–matrix remodeling: regulation of actin cytoskeleton, focal adhesion, and ECM–receptor interaction. These results confirmed previous findings that in cancer the ECM matrix is subject to dynamic changes that reflect progression and metastasis [47,48]. Together with collagens and laminins, ECM matrix modification stimulated cancer cell activity and tumor progression [49,50]. The present study allowed us to identify the differential expression of collagens, laminin, and integrins. The detected significantly enriched GO was due to the collagen fibril organization. The collagen family is the most exposed DEG family in this analysis, in contrast to the analysis performed for the BPV-E4 protein. The integrin and kinesin superfamily proteins had the largest share. Moreover, both *BPV-E4*- and *BPV-E1^E4*-mediated nucleofection of equine ACFCs brought about the upregulation of *FGFR3* and *FGF12* (fibroblast growth factor receptor 3 and fibroblast growth factor 12), which are known as factors promoting tumor growth and metastasis [51].

Surprisingly, we have also observed differential expression of the *F2R* gene (encoding coagulation factor II thrombin receptor), which, according to the literature, can stimulate the migration and invasion of cancer cells under *SOX9* influence [52]. The other gene upregulated in nucleofected equine ACFCs was *ROCK2* (Rho associated coiled-coil containing protein kinase 2). Kaczorowski et al. [53] indicated that both *ROCK1* and *ROCK2* genes can be critical for controlling cellular motility and cancer invasiveness, while the inhibition of *ROCK2* decreased the tumor growth based on the osteosarcoma model [54].

The equine ACFCs nucleofected with the *BPV-E1^E4* gene construct displayed significant deregulation of s higher number of pathways than *BPV-E4* transgenic ACFCs, such as the FoxO signaling pathway, the PI3K-Akt and *TNF* signaling pathways, and Proteoglycans in cancer. The study by Semik et al. [16], which was focused on transcriptome differences between sarcoid and healthy skin tissues, showed significant over-representation of genes belonging to the PI3K-Akt signaling pathway, pathways in cancer, and cytokine–cytokine receptor interaction. Moreover, the abovementioned authors have observed differences in the expression of genes involved in actin cytoskeleton regulation, tight junction, and cell adhesion. Genes with differential expression such as *FGFR2* and *FGF10* have been identified in healthy and sarcoid-related tissues [16] and, analogously, in both *BPV-E1^E4* transgenic ACFCs and their control, nontransgenic counterparts. Similar to in the present study, healthy skin and sarcoids were characterized by differences with regard to collagens, integrins, and tubulin genes, which can affect the cytoskeleton arrangement and cell mobility [16].

Interestingly, in the current in vitro study, we noticed the significant downregulation of the *IGF2* (insulin-like growth factor 2) and *EGFR1* (epidermal growth factor receptor 1) genes. Such results are in contrast to the literature data, which showed overexpression of both genes in different types of cancers. The increased expression of the *IGF2* gene is strongly associated with a poor prognosis via stimulating cell proliferation [55]. Similarly, upregulation of the *EGFR1* gene, which is closely related to the tumor stage [56], and its overexpression means a poor prognosis of clinical outcome [57]. The low expression of both genes is characteristic for normal cells, but not for their neoplastic counterparts. Nonetheless, *EGFR1* can be downregulated by different factors such as decorin [58] or ubiquitin-specific peptidase 8 (*UBPY*) [59]. Such a mechanism aims to terminate cell proliferation and stop uncontrolled cell growth, which contributes to carcinogenesis. On the other hand, we observed the significant upregulation of the insulin receptor gene (*ISNR*), which is responsible for stimulation of tumor cell proliferation, migration, and invasion [60], and can be co-expressed with the *EGFR* gene in tumors [61]. Overexpression of *INSR* is correlated with a poor prognosis for oncology patients [62] and can be used as an early tumor-related marker [63]. However, it should be highlighted that, in this study, the effect of only one gene of the *BP* virus is investigated. The aforementioned differences in the achieved gene expression levels may result from the lack of interaction with other viral proteins. Thus, neoplastic changes may occur differently.

To sum up, the results of the current investigation have confirmed that *BPV-E4*- and *BPV-E1^E4*-mediated nucleofection significantly affected transcriptomic alterations, leading to sarcoid-like neoplastic transformation of equine ACFCs. Nevertheless, the changes in transcriptomic signatures arising in the cells nucleofected with *BPV-E1^E4* fusion genes increasingly tended to resemble those that occurred in vivo in equine sarcoids. This finding may be justified by the onset and progression of modifications in crucial signaling pathways such as PI3K-Akt-mediated signal transduction pathway and a variety of closely related pathways. For this reason, we propose the strategy based on transgenically induced expression of BPV-E1^E4 fusion protein as a completely new ex vivo model of sarcoid-dependent oncogenic transformation in equine ACFCs. This biomedical model can be used not only to more comprehensively explore and decipher the molecular nature of dermal sarcoid-like neoplasia, but also to preclinically or clinically predict the directions and targets of anticancer therapies in specimens afflicted with epidermal and dermal sarcoids.

## 4. Materials and Methods

### 4.1. Experimental Schedule

**Figure 7:**
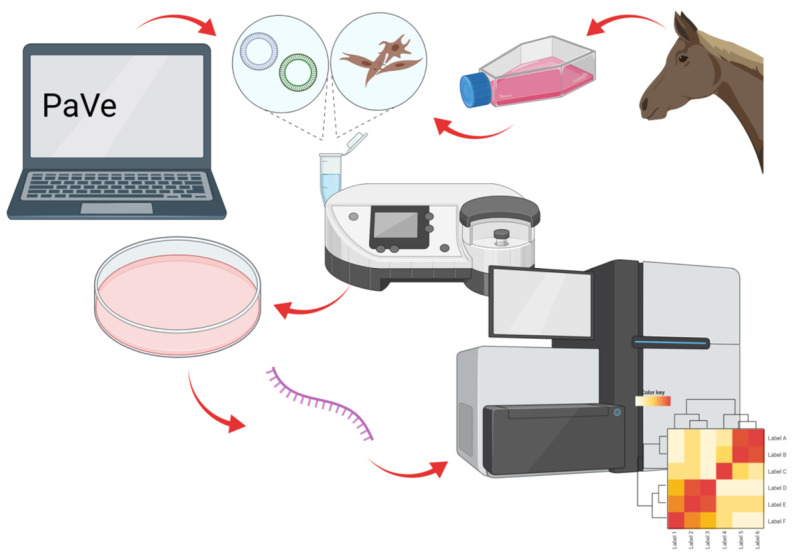
Experimental schedule. Created with BioRender.com [64].

The experimental protocol (as depicted in Figure 7) was divided into three main steps: (1) designing and preparing the transgene sequences to be expressed in equine ACFCs; (2) nucleofection-mediated neoplastic transformation of ACFCs prompted by *BPV-E4* and *BPV-E1^E4* transgenes; and (3) analysis of transcriptome changes in *BPV-E4* and *BPV-E1^E4* transgenic cells.

In the first series of experiments, the gene sequences were designed based on information available in the biological database PaVe [65]. The sequences were cloned by the manufacturer (GeneArt Gene Synthesis, Thermo Scientific, Waltham, MA, USA) into pMA-T plasmids, from which they were cut out with appropriately selected restriction enzymes. The excised sequences were cloned into expression plasmids (T-REx System, Invitrogen, Waltham, MA, USA, Thermo Scientific). The plasmids were propagated in competent *Escherichia coli* bacteria (strain DH5α; Invitrogen).

In the second series of experiments, the sarcoid-dependent genetic transformation of ACFCs was induced by nucleofection with the use of *BPV-E4* and *BPV-E1^E4* transgenes. Positively transformed nucleofectants that had acquired combined immune resistance to a cocktail of select antibiotics were expanded ex vivo and subsequently assigned to a further series of experiments.

In the third series of experiments, in order to perform a transcriptome analysis, RNA samples were isolated from the control (nontransgenic) and *BPV-E4* and *BPV-E1^E4* transgenic cell lines, from which cDNA libraries were then derived. The assessment of transcriptomic profiles was accomplished by next-generation sequencing (NGS) on an Illumina apparatus (Illumina, San Diego, CA, USA).

### 4.2. Designing Gene Inserts for Further Experiments Aimed at Nucleofection of Equine ACFCs

The inserted sequence of the *BPV-E4* gene was designed on the basis of the information available in the papillomavirus database, PaVe [65]. The sequence of the analyzed gene was designed with two variants. The first variant was based on the amino acid sequence of the *BPV1-E4* protein (gi 60965.E4) transcribed into the sequence encoding a given gene. The second variant was based on the amino acid sequence of the *BPV-E4* protein, including the amino acid sequence of the *BPV-E1* protein fragment (gi 60965), which more closely corresponds to the actual structure of the *BPV-E4* protein in vivo. In addition, the sequences were flanked with amino acid sequences characteristic of restriction enzymes (two different enzymes for each insert) enabling the creation of sticky ends. The enzymes were selected based on the MCS sequence of the plasmids of the target inserts (*pcDNA4/TO/myc-his/B*; T-REx System; Invitrogen) in such a way that the sequence ends they formed were not complementary. Such selection of enzymes prevented the formation of circular structures inside the enzymatic digestion products and also ensured that the insert sequence was placed in the correct direction concerning the target plasmid sequence. Additionally, the sequences of the inserts were enriched with the consensus KOZAK sequence (gccgccaccatgg).

### 4.3. The Reactions of Enzymatic Restriction and Ligation

The insert sequences provided by the manufacturer were cloned into *pMA-T* plasmids, from which they were excised using the restriction enzymes included in the design process. The reaction mixture contained a DNA template in the form of a plasmid containing the appropriate gene and a set of specific enzymes along with a buffer matched to them (for the gene: *BPV-E4*-AflII, KpnI, buffer 2.1; *BPV-E1^E4*-SacII, AflII, Cut Smart buffer; New England BioLabs, Ipswich, MA, USA), and digestion was carried out overnight. In addition, pcDNA plasmids from the T-REx system set (*pcDNA4/TO/myc-His/B*; Invitrogen) were also digested by restriction enzymes corresponding to the individual sequences of the inserts. The amount of template DNA was estimated to obtain 400 ng of the actual product (cut insert sequence or linear plasmid), which corresponds to the maximum amount of DNA that could be used in one sample during the gel purification method, made in the next step.

Digestion products were separated with agarose gel electrophoresis (0.8% low melting point agarose in TBE buffer, 80 V, until DNA band separation). The DNA band containing the viral gene sequence (*BPV-E4* or *BPV-E1^E4*) or a linear form of the digested plasmid was cut from the gel (ethidium bromide-mediated staining) and purified with a High Pure PCR Product Purification Kit (Roche, Warsaw, Poland). Purified DNA was eluted in the manufacturer’s buffer, heated to 56 °C.

The obtained fragments of the corresponding gene variants were combined with the *pcDNA 4/TO/myc-His/B* plasmid in a mass ratio of 3:1. The required volumes of individual DNA were calculated using an online calculator [66]. According to the manufacturer’s guidelines, the reaction was performed with a Rapid DNA Ligation Kit (Roche, Basel, Switzerland).

### 4.4. Molecular Cloning of DNA Plasmid Constructs with Inserted BPV-E4 or BPV-E1^E4 Gene Sequences

Plasmids were cloned with Subcloning Efficiency DH5α Competent Cells (Invitrogen). Ten nanograms of plasmid DNA were introduced into bacteria by the heat-shock method. After the addition of DNA, the bacteria were held at 42 °C for 20 s after 30 min of incubation on ice, and then the bacteria were put on ice again for 2 min. Transformed bacteria were incubated in 1 mL low-salt Luria-Bertani Broth (Sigma-Aldrich, Merck Life Sciences, Poznań, Poland) for 1 h at 225 rpm and 37 °C. The bacteria were seeded on a low-salt LB broth with the addition of agarose (Sigma-Aldrich) and 120 µg/mL ampicillin (Gibco, Thermo Scientific), which served as a selective antibiotic, and incubated overnight at 37 °C. The genetically transformed bacterial cells that had been found to display immune resistance to ampicillin were positive for the occurrence of plasmid DNA. Obtained bacterial colonies were tested for positive recombination with Quick Screen PCR. Fragments of picked bacterial colonies were suspended in 15 µL of 0.1% Triton X-100 (Sigma-Aldrich) in TE buffer, then incubated in 100 °C for 5 min and centrifuged (13,000× *g*; 10 min). The supernatant was sequenced (Sanger method; Genetic Analyzer XL, Applied Biosystems, Thermo Fisher Scientific) to confirm the presence and quality of plasmids. Bacterial colonies that were positive for plasmid presence were grown for 14 h at 37 °C (250 rpm) in 100 mL of low-salt LB broth (Sigma-Aldrich) enriched with 120 µg/mL ampicillin. Suspended bacteria were centrifuged (4500× *g*; 20 min; 4 °C) followed by removal of supernatants. Plasmid DNA was isolated with a Qiagen Plasmid Midi Kit (Qiagen, Wroclaw, Poland), according to the manufacturer’s protocol. Plasmid DNA was eluted in 50 mL of TC-treated water.

### 4.5. Establishment of Primary Cultures and Mitotically Stable Lines of Equine Adult Cutaneous Fibroblast Cells (ACFCs)

Adult skin tissue-derived biopsies (*n* = 4) were collected postmortem from the lower eyelid regions of horses slaughtered in the local abattoir. Dermal tissue samples were deposited into tubes filled with Dulbecco’s Modified Eagle’s Medium (DMEM; Gibco) supplemented with 10% fetal bovine serum (FBS; Gibco), HEPES (Gibco) and primocin (InvivoGen, Alab, Warsaw, Poland). Tubes were stored at 4 °C for no longer than 24 h after the recovery of cutaneous tissue explants.

Primary cell cultures were generated according to the modified procedures described in the study by Tomasek et al. [67]. Briefly, dermal tissue samples were disinfected with 70% ethanol and washed thrice in a 1× solution of Dulbecco’s phosphate-buffered saline (DPBS; pH 7.2; Gibco), followed by cutting into smaller pieces (approximately 2 mm × 2 mm), which were placed into cell culture flasks containing DMEM (Gibco) enriched with 10% FBS and primocin. Cutaneous tissue fragments were incubated at 37 °C in an atmosphere of 5% CO_2_ and 100% humidity for two weeks, until the cells spontaneously migrated from the tissue explants. The culture medium was changed two times per week and passages were performed immediately after the ex vivo proliferating cells had reached 90% confluence. The first passages were characterized by the presence of epidermal keratinocytes in culture. Therefore, during the passaging procedure, the cells were trypsinized until the adherent equine adult cutaneous fibroblast cells (ACFCs) were efficiently detached. Keratinocytes, as less detachable epidermal cells [68], were still attached to the bottom of the culture dishes. For that reason, these cell subpopulations have not been replated. The homogenous ACFC lines, in the subpopulations of which the disappearance of epidermal keratinocytes was clearly confirmed, were successfully established at the third passage.

### 4.6. Genetic Transformation of Equine ACFCs Mediated by Nucleofection

The approaches that were applied both to prepare the equine ACFCs prior to nucleofection and to nucleofect them were accomplished according to the methods used for the transgenization of porcine dermal fibroblast cells (NDCs for SCNT), as comprehensively described in studies by Skrzyszowska et al. [21] and Samiec et al. [22]. Briefly, the ex vivo expanded equine ACFCs that had previously reached approximately 90% confluence were prepared for the nuclefection procedure by trypsinization and subsequent resuspension in HEPES-buffered Tissue Culture Medium 199 (TCM 199; Sigma-Aldrich) supplemented with 5% FBS (Sigma-Aldrich), followed by centrifugation at 200× *g* for 10 min. Afterwards, the centrifugation pools of cells (each at a concentration ranging from 4 × 10^5^ to 5 × 10^5^) were subjected to co-transfection nucleofection using the Amaxa^TM^ Normal Human Dermal Fibroblast– Adult (NHDF-Adult) Nucleofector^TM^ Kit (Lonza, CELLLAB, Warsaw, Poland) and a mixture of two pcDNA plasmids included in the T-REx kit (Invitrogen). The aforementioned mixture of two plasmids (a total amount of 2.8 µg and in a mass ratio of 6:1) was comprised of pcDNA™ *6/TR* and pcDNA™ *4/TO/myc-His/B* with the appropriate transgene variant inserted (either *BPV-E4* or *BPV-E1^E4*). The co-transfection of equine ACFCs was carried out within the Amaxa nucleofection cuvettes inserted into the holder of the Amaxa Nucleofector^TM^ II Device (Amaxa Biosystems, Lonza, Medianus, Kraków, Poland). The nucleofection process was triggered by the U-023 program intended for transgenization of human dermal fibroblasts and resulted in high transfection efficiency. The U-023 program was delivered by Amaxa Nucleofector^TM^ Technology (Amaxa Biosystems).

### 4.7. Treatment of Cell Nucleofectants Leading to Positive Antibiotic-Dependent Selection of BPV-E4 or BPV-E1^E4 Transgenic Equine ACFCs and Their Subsequent Tetracycline-Induced Neoplastic Transformation into Sarcoid-like Cells

After nucleofection, the equine ACFCs were seeded into collagen-coated culture dishes (Greiner Bio-One GmbH, BIOKOM Systems, Janki near Warsaw, Poland) and incubated for 48 h in DMEM enriched with recombinant human basic fibroblast growth factor (rh-bFGF; Sigma-Aldrich). The culture medium was subsequently changed to a medium supplemented with 200 µg/mL zeocin (Invitrogen) and 6 µg/mL blasticidin S (Thermo Scientific, Waltham, MA, USA). As a consequence of zeocin- and blasticidin S-dependent negative selection, the nontransgenic (TG^–^) cells that had not effectively undergone *BPV-E4*- or *BPV-E1^E4*-induced oncogenic transformation were eliminated from subpopulations encompassing cell nucleofectants due to the lack of immune resistance to selective antibiotics (i.e., combined resistance to zeocin and blasticidin S). The ACFCs that had undergone efficient transgenization were found to display antibiotic resistance. Seven days later, the selection was complete and the remaining positively selected transgenic (TG^+^) cells were cultured under standard conditions in the medium supplemented with 10% Tet-System Approved FBS (Gibco). Plasmid expression was induced by the addition of 1 µg/mL tetracycline (Invitrogen) to the culture medium 24 h before accomplishing further procedures.

The concentrations of the individual antibiotics were selected by establishing the lowest concentrations of the antibiotics that destroyed the cell culture within a week. For this purpose, media with different concentrations of individual antibiotics were introduced into the cultures, carried out in 96-well culture plates with 100% confluence. The concentrations were 2, 4, 6, 8, 9, 10, 11, 12, and 14 µg/mL for blasticidin S and 50, 100, 200, 400, 600, 800, 1000, 1100, and 1300 µg/mL for zeocin. One week later, the number of vial cells was measured using CellTiter Blue dye (Promega, Walldorf, Germany). The culture medium was removed from each well, and then 100 µL of culture medium with dye was added to it (in a 5:1 ratio). The cultures were then incubated for 5.5 h in an incubator (37 °C, 5% CO_2_, 100% humidity), protected from light. The measurement was performed on a PlateReader 2200 (Eppendorf, Warsaw, Poland) (excitation: 535 nm, emission: 595 nm). The lowest concentrations of antibiotics were selected as those for which the fluorescence level did not differ significantly from the fluorescence of empty wells.

### 4.8. Detection of BPV DNA in Equine ACFCs Subjected to Oncogenic Transformation with the Aid of Nucleofection

DNA was isolated with a NucleoMag Vet Kit (Macherey-Nagel, Bionovo, Legnica, Poland) according to the manufacturer’s protocol. DNA was dissolved in DEPC-treated water (Life Technologies, Ambion, Thermo Scientific, Waltham, MA, USA). The quality of isolated DNA was checked with NanoDrop 2000 (Life Technologies).

Polymerase chain reaction (PCR) was performed with AmpliTaq Gold 360 Master Mix polymerase (Applied Biosystems) with primers specific for the *BPV1* and *BPV2* consensus region [69]. The temperature profile was set with respect to the polymerase supplier’s protocol and with a primer annealing temperature of 57–58 °C. PCR products were separated in agarose gel electrophoresis (3% agarose in TBE).

### 4.9. Isolation of RNA Samples from BPV-E4 and BPV-E1^E4 Transgenic Equine ACFC-Derived Neoplastic Cells

According to the producer’s protocol, RNA was directly isolated from adherent cultures of *BPV-E4* and *BPV-E1^E4* transgenic equine ACFC-derived neoplastic cell lines that were expanded ex vivo on the bottom of culture dishes. To extract RNA samples, a PureLink™ RNA mini kit (Invitrogen) was used. The procedure was maintained, with the addition of a DNase treatment step (PureLink™ DNase Set, Invitrogen). RNA was eluted with DEPC-treated water (Thermo Fisher Scientific, Waltham, MA, USA).

The quality and quantity of RNA were measured with a 2200 TapeStation Automated Electrophoresis System (Agilent Technologies, Santa Clara, CA, USA), as well as a nanodrop 2000 spectrophotometer (Thermo Scientific) and agarose gel (2% agarose in TBE buffer) electrophoresis.

### 4.10. NGS Sequencing among Oncogenically Transformed Equine ACFCs Expressing BPV-E4 and BPV-E4^E1 Transgenes

All samples were sequenced using the NGS approach. High-quality RNA (RIN value from 9.3 to 9.8) was used for cDNA libraries preparation with the TruSeq RNA Kit v2 kit (Illumina, San Diego, CA, USA) according to the attached protocol. The individual cDNA libraries were ligated with different indexes to be able to pool samples during the NGS sequencing procedure. The quality and quantity of obtained libraries were assessed using Qubit 2.0 (Qubit™ dsDNA BR AssayKit, Invitrogen, Waltham, MA, USA) and TapeStation 2200 (D100 ScreenTapes, Agilent Technologies, Santa Clara, CA, USA). In the next step, the cDNA libraries were sequenced on the NextSeq 500 Illumina platform (Illumina) and NextSeq 500/550 High Output KIT v 2.5 (75 cycles) according to the protocol.

The raw data were first checked for quality with FastQC v0.11.7 software, followed by the removal of adapters and low-quality reads (Phred quality of 20 and read length of 36). Then, the filtered reads were mapped to the EquCab3 genome with STAR software. Afterwards, the mapped reads were annotated and counted to specific gene thresholds with the usage of Ensembl GTF file version 100 (via htseq-count software). Differential expression analysis was performed with the use of Deseq2 software v3.14.

Gene Ontology enrichment and over-represented Pathways analyses (KEGG, GO) were performed using David software (version 6.8) [70] based on the *Equus caballus* reference. The significance was based on the False Discovery Rate (FDR), calculated as a *p*-value after Benjamin multiple testing correction [71]. For the visualization of gene interaction, String software v11.5 [24] was applied with *Equus caballus* as a reference.

### 4.11. qPCR-Assisted Validation Accomplished for Transcriptional Activity Levels of Genes in Neoplastically Transformed Equine ACFCs Expressing BPV-E4 and BPV-E1^E4 Transgenes

RNA-seq validation was performed using real-time PCR. The exact transcript levels were estimated for 10 DEGs for which specific primers were designed based on Ensemble reference (Primer3 Input (version 0.4.0) software; [72]) (Table S1). The cDNA samples were synthesized from 300 ng of total RNA using a High-Capacity RNA-to-cDNA™ Kit (Applied Biosystems). The qPCR reaction was carried out in triplicate for each sample with Sensitive RT HS-PCR EvaGreen Mix (A&A Biotechnology, Gdynia, Poland) according to the manufacturer’s protocol and using QuantStudio7Flex platform (Applied Biosystems). The expression was calculated using the delta–delta CT method, according to Pfaffl et al. [73], and based on two endogenous controls, i.e., *ACTB* and *UBB* genes that encode β-actin and ubiquitin B, respectively [74].

The NGS data (normalized counts) and relative quantity (RQ) were compared using the Pearson correlation (SAS software, version 8.02).

## 5. Conclusions

Our research sought to unravel the transcriptomic signatures of in vitro proliferating *BPV-E4* and *BPV-E4^E1* transgenic equine ACFCs that have undergone sarcoid-dependent oncogenic transformation. It might contribute to further investigations focused on somatic cell cloning in domestic horses and other equids. The goal of these investigations might be determining the suitability of nonmalignant sarcoid-like derivatives of ACFCs to be used as an epigenomically plastic and dedifferentiable source of NDCs for generating equine cloned embryos and offspring by SCNT-mediated ARTs. This might be of importance for both empirically and preclinically developing novel ex vivo biomedical models. The latter will attempt to track and decipher the molecular pathways of the processes responsible for the epigenomic reprogrammability of transcriptional profiles within the nuclear DNA of transgenic equine ACFC-derived sarcoid-like cells. On the one hand, the formerly indicated processes have been found to incur at the onset and progression of sarcoid-dependent neoplasia due to nucleofection-mediated cancerous transformation under extracorporeal conditions. On the other hand, these processes might trigger the irreversible attenuation of neoplastic transformation into dermal sarcoid-like tumors and subsequent initiation of the anticancer conversion of neoplastic ACFCs as a result of SCNT-based cloning, not only in horses but also in other taxonomic representatives of the *Equidae* family.

## Figures and Tables

**Table 1:** The significant enrichment in Gene Ontology terms detected on the basis of comparative analysis of DEGs set between *BPV-E4* and the control group.

Gene Ontology	N_all_	N_u_	Upregulated Genes	N_d_	Downregulated Genes	FDR
positive regulation of cell migration	24	14	*SEMA4D, CORO1A, INSR, F2R, DAB2, PDGFD, MMP14, SDCBP, CDH13, PIK3R1*	10	*ATP8A1, COL18A1, ARHGEF39, CCL26, HAS2, SEMA7A, BMP2, SNAI1, EGFR, TRIP6*	<0.001
negative regulation of cell proliferation	34	17	*IFIT3, SKAP2, IRF1, F2R, SMAD1, KAT2B, ZEB1, TESC, GLI3, CDH13*	17	*EREG, CER1, FEZF2, WNK2, AXIN2, HMGA1, RBPJ, PTPN14, SPRY2, BMP2*	<0.001
cell-matrix adhesion	15	5	*VCAM1, SNED1, ITGB4, CL2L11, ITGA8*	10	*EPDR1, ITGB6, TECTA, OTOA, FREM1, TNN, STRC, ITGB3, ITGA6, ITGA1*	<0.001
cell migration	21	9	*SDC4, ASAP3, RASGEF1A, JAK2, LIMD1, MMP14, CLN3, JAK1, NDE1*	12	*TNS3, DEPDC1B, TNN, HES1, CSPG4, ELMO1, SDC1, SNAI1, ABL2, FSCN1*	<0.001
mitotic spindle assembly	9	3	*KIF3B, WRAP73, ARHGEF10,*	6	*BIRC5, NEK2, MYBL2, KIFC1, KIF11, RAB11A*	0.002
mitotic cytokinesis	8	0	*-*	8	*NUSAP1, KIF20A, CEP55, KIF23, RACGAP1, ANLN, CKAP2, PLK1,*	0.002
chromosome segregation	11	2	*NDE1, NEK3*	9	*NEK2, HJURP, CENPT, SPC25, CENPN, KIF11, CENPW, CDCA2, RCC1*	0.002
actin cytoskeleton organization	15	8	*CDC42, EP2, CORO1A, RHOJ, SDCBP, NISCH, CLN3, WASF2, BCL6*	7	*ARHGAP26, ELMO1, DIAPH3, NUAK2, ABL2, PFN1, TMSB4X*	0.002
cell adhesion	21	5	*GPNMB, ITGA8, CERCAM, EPHB4, TNFAIP6*	16	*POSTN, TNC, TGFBI, COL18A1, NINJ1, HES1, SUSD5, HAS2, ITGA6, COL15A1*	0.003

N_all_—Number of all detected DEGs; N_u_—Number of upregulated DEGs; N_d_—Number of downregulated DEGs; FDR—False Discovery Rate in DAVID software.

**Table 2:** The significant enrichment in Gene Ontology terms detected on the basis of comparative analysis of DEGs set between *BPV-E1^E4* and control group.

Gene Ontology	N_all_	N_u_	Upregulated Genes	N_d_	Downregulated Genes	FDR
negative regulation of canonical Wnt signaling pathway	33	21	*EGR1, WNT5A, DKK2, SOX9, LIMD1, GREM1, BICC1, GLI3, STK4, LATS1*	12	*NOTUM, WNT11, GPC3, DRAXIN, AXIN2, CAV1, LRP4, NPHP4, MLLT3, MAD2L2*	0.009
focal adhesion	99	65	*ITGA8, SORBS1, CNN1, MCAM, ITGA11, SYNPO2, FBLN7, NEXN, LPP, PHLDB2*	34	*CSPG4, PROCR, FLRT2, HMGA1, TNS4, TSPAN4, CAV1, FHL1, KIF22, PLAU*	
negative regulation of extrinsic apoptotic signaling pathway	15	13	*TGFBR1, COL11A1, COL1A1, LOX, COL5A1, GREM1, P4HA1, LOXL2, COL1A2, NF1*	2	*FMOD, ANXA2*	<0.001
transforming growth factor beta receptor signaling pathway	19	12	*TGFBR1, FOS, SKIL, SMAD4, SMURF1, COL1A2, SMAD9 FERMT2, TGFBR3, MTMR4,*	7	*HPGD, SMAD6, PTPRK, SMURF2, TAB1, PXN, TGFB3*	<0.001
collagen fibril organization	15	13	*TGFBR1, COL11A1, COL1A1, LOX, COL5A1, GREM1, P4HA1, LOXL2, COL1A2, NF1*	2	*FMOD, ANXA2*	<0.001

N_all_—Number of all detected DEGs; N_u_—Number of upregulated DEGs; N_d_—Number of downregulated DEGs; FD—False Discovery Rate in DAVID software.

**Table 3:** The significant enrichment in molecular KEGG pathways detected on the basis of comparative analysis of DEGs set between *BPV-E4* and control group.

KEGG Pathways	N_all_	N_u_	N_d_	FDR	Most Deregulated Genes	
					Up	Down
Focal adhesion (ecb04510)	31	11	20	0.051	*ITGB4, LAMA3, XIAP, ITGA8, PDGFD, PIK3R1, SOS2, ROCK2, LAMB2, ERBB2*	*TNC, ITGB6, CCND2, TNN, CCND1, COL6A6, SHC3, ACTN3, ITGB3, BAD*
Regulation of actin cytoskeleton (ecb04810)	34	13	21	0.008	*FGF18, ITGB4, F2R, ITGA8, ARHGEF6, PDGFD, DIAPH2, PIK3R1, SOS2, ROCK2*	*ITGB6, FGF12, BDKRB2, IQGAP3, FGFR3, ACTN3, ITGB3, DIAPH3, ITGB7, ITGA6*
ECM-receptor interaction (ecb04512)	19	5	14	0.010	*ITGB4, LAMA3, SDC4, ITGA8, LAMB2*	*TNC, ITGB6, TNN, COL6A6, HMMR, ITGB3, ITGB7, ITGA6, SDC1, ITGA1*
PI3K-Akt signaling pathway (ecb04151)	44	20	24	0.051	*FGF18, ITGB4, LAMA3, INSR, CREB3L1, BCL2L11, F2R, TGA8, NR4A1, JAK2*	*TNC, ITGB6, FGF12, CCND2, TNN, CCND1, ANGPT1, COL6A6, FGFR3, ITGB3*
Cell cycle (ecb04110)	28	9	18	0.001	*CDC14A, RB1, STAG1, CDC25B, E2F5, SMC3, RBL1, RBL2, RAD21*	*CCND2, CDC45, CCND1, MCM5, CCNB2, CCNB1, CDC20, E2F1, CDK1, BUB1*
Steroid biosynthesis (ecb00100)	9	1	8	0.008	*SOAT1*	*HSD17B7, TM7SF2, LSS, SQLE, FDFT1, SQLE, FDFT1, FAXDC2, EBP, SC5D*
Pathways in cancer (ecb05200)	52	23	29	0.010	*FGF18, LAMA3, FOS, XIAP, F2R, TGFBR2, ADCY9, MITF, RB1, ADCY3*	*CTNNA2, WNT7B, FGF12, MMP1, CXCL8, BDKRB2, TCF7, BIRC5, CCND1, AXIN2*

N_all_—Number of all detected DEGs; N_u_—Number of upregulated DEGs; N_d_—Number of downregulated DEGs; FD—False Discovery Rate in DAVID software.

**Table 4:** The significant enrichment in molecular KEGG pathways detected on the basis of comparative analysis of DEGs set between *BPV-E1^E4* and control group.

KEGG Pathways	N_all_	N_u_	N_d_	FDR	Most Deregulated Genes	
					Up	Down
Focal adhesion (ecb04510)	63	42	21	<0.001	*ITGA8, THBS1, ITGA11, OL11A1, XIAP, PDPK1, LAMA3, MYLK3, ROCK2, PP1R12A*	*LAMC3, SHC3, COL5A3, COL4A1, CCND1, CCND2, COL6A6, BAD, VEGFD, COL6A3*
Regulation of actin cytoskeleton (ecb04810)	57	43	14	<0.001	*FGF21, ITGA8, FGF5, ITGA11, MYLK3, ROCK2, PPP1R12A, PFN2, FGFR2, ARHGEF6*	*BDKRB2, FGFR3, ITGAX, FGF12, IQGAP2, DIAPH3, ITGA6, GSN, ITGAE, EGFR*
ECM-receptor interaction (ecb04512)	30	18	12	0.001	*ITGA8, THBS1, ITGA11, COL11A1, LAMA3, LAMA5, COL1A1, COL5A, ITGB7, FN1,*	*LAMC3, COL5A3, COL4A1, COL6A6, COL6A3, ITGA6, SDC1 CD44, AGRN, TNN,*
PI3K-Akt signaling pathway (ecb04151)	73	47	26	0.019	*FGF21, ITGA8, FGF5, THBS1 ITGA11, COL11A1, CREB3L1, INSR, EFNA1, DPK1*	*IL6LAMC3, FGFR3, COL5A3, COL4A1, CCND1, FGF12, CCND2 IL7, COL6A6,*
Cell cycle (ecb04110)	32	10	22	0.047	*GADD45B, RBL1, SMAD4, STAG1, EP300, CDC27, AD21, YWHAG, STAG2, E2F5*	*CCND1, CCND2, CCNB2, CDC20, MCM5, CCNB1, CDK1, CDC45 PKMYT1, PLK1,*
FoxO signaling pathway (ecb04068)	38	22	16	0.003	*TGFBR1, INSR, PDPK1, PRKAB2, AKT3, FBXO32, IRS2, GADD45B, SMAD4, PRKAG3*	*IL6, CCND1, CCND2, CCNB2, CCNB1, TNFSF10, S1PR1, PLK1, CDKN2B, G6PC3,*
Proteoglycans in cancer (ecb05205)	48	27	21	0.019	*ITPR1, THBS1, WNT5A, PDPK1, ROCK2, PPP1R12A, AKT3, FN1, CAMK2D, PIK3R1*	*WNT11, ERBB3, GPC3, CCND1, WNT7B, HPSE, MMP9, TIMP3 CAV1, IGF2,*
Rap1 signaling pathway (ecb04015)	49	34	15	0.035	*FGF21, FGF5, THBS1, INSR, ADCY5, EFNA1, AKT3, SIPA1L2, PFN2, ADCY9*	*RAP1GAP, FGFR3, FGF12, ID1, ADORA2A, VEGFD, ANGPT1, ANGPT4, HGF, MAP2K3,*
TNF signaling pathway (ecb04668)	29	16	13	0.047	*MAP3K8, EDN1, CREB3L1, FOS, AKT3, CREB3L2, TAB3, PIK3R1, ITCH, MAP3K5*	*CSF2, IL6, CXCL1, VCAM1, MMP9, IL15, CREB3L4, MAP2K3, CCL2, TRADD*

N_all_—N_u_—Number of upregulated DEGs; N_d_—Number of downregulated DEGs; FDR—False Discovery Rate in Number of all detected DEGs; DAVID software.

**Table 5:** The correlation coefficients and their corresponding *p*-value for qPCR validation.

Gene	Accession Number	Correlation Coefficient
*MMP2*	ENSECAG00000000953	0.839 ***
*MMP14*	ENSECAG00000008351	0.887 *
*MMP9*	ENSECAG00000013081	0.662 *
*MMP15*	ENSECAG00000000196	0.897 **
*MMP17*	ENSECAG00000013201	0.440 ^ns^
*MMP24*	ENSECAG00000024778	0.814 *
*PTGER2*	ENSECAG00000009713	0.686 *
*TIMP1*	ENSECAG00000014259	0.989 ***
*FGF10*	ENSECAG00000014361	0.748 *
*RECK*	ENSECAG00000010426	0.688 *

* *p*-value < 0.05; ** *p*-value < 0.001; *** *p*-value < 0.0001; ns—nonsignificant.

## Data Availability

The RNA-seq data were submitted to the GEO database and are available under GSE193906 accession number.

## Supplementary Materials

The following supporting information can be downloaded at https://www.mdpi.com/article/10.3390/ijms23041970/s1.

## Abbreviations

ACFC	Adult cutaneous fibroblast cell
ACTB	Actin β
ADAM	Disintegrin and metalloproteinase domain
Akt	Serine/threonine kinase
ART	Assisted reproductive technology
BAD	Bcl2-associated agonist of cell death
BCG	Bacillus Calmette–Guérin
BMI	Polycomb ring finger
BPV	Bovine Papillomavirus
CCND	Cyclin D
CD	Cluster of differentiation
CDK	Cyclin-dependent kinase
COL	Collagen
DAVID	Database for Annotation, Visualization, and Integrated Discovery
DEG	Differentially expressed gene
DMEM	Dulbecco’s Modified Eagle’s Medium
DPBS	Dulbecco’s phosphate-buffered saline
ECM	Extracellular matrix
EGFR	Epidermal growth factor receptor
F2R	Coagulation factor II thrombin receptor
FBS	Fetal bovine serum
FDR	False discovery rate
FGF	Fibroblast growth factor
FGFR	Fibroblast growth factor receptor
FOS	Fos proto-oncogene
FoxO	Forkhead box O
GEO	Gene Expression Omnibus
GO	Gene Ontology
HEPES	4-(2-hydroxyethyl)-1-piperazineethanesulfonic acid
IGF	Insulin-like growth factor
ISNR	Insulin receptor
ITG	Integrin
ITGA	Integrin subunit α
KEGG	Kyoto Encyclopedia of Genes and Genomes
KIF	Kinesin superfamily protein
LAMA	Laminin subunit α
LB	Luria–Bertani
MAPK	Mitogen-activated protein kinase
MCS	Multiple cloning site
MMP	Matrix metalloproteinase
NANOG	Nanog homeobox
NCR	Noncoding region
NDC	Nuclear donor cell
NGS	Next generation sequencing
PCA	Principal component analysis
PCR	Polymerase chain reaction
PI3K	Phosphoinositide 3-kinase
PTGER	Prostaglandin E receptor
Rap	Ras-related protein
RECK	Reversion inducing cysteine rich protein with kazal motifs
Rho	Rhodopsin
ROCK	Rho-associated coiled-coil containing protein kinase
SCNT	Somatic cell nuclear transfer
SOX	SRY-box transcription
TBE	Tris/borate/EDTA
TCM	Tissue culture medium
TE	Tris-EDTA
TGFB	Transforming growth factor β
TGFBR	Transforming growth factor β receptor
TIMP	Tissue inhibitor of metalloproteinase
TNF	Tumor necrosis factor
UB	Ubiquitin
UBP	Ubiquitin-specific peptidase
URR	Upstream regulatory region
VEGF	Vascular endothelial growth factor
Wnt	Wingless-type MMTV integration site family
XIAP	X-linked inhibitor of apoptosis
