# Supplementary material for: The Induced Expression of BPV E4 Gene in Equine Adult Dermal Fibroblast Cells as a Potential Model of Skin Sarcoid-like Neoplasia
Source: Int J Mol Sci. 2022 Feb 10;23(4):1970. doi: 10.3390/ijms23041970 (PMC8877312; doi:10.3390/ijms23041970)
Supplement: Supplementary file 1 [file ijms-23-01970-s001.zip › Supplementary Table S1 Real Time PCR Primer Sequences.pdf]

Supplementary Table S1. The primer sequences used in real-time PCR analysis.

Gene Name	***Accesion Number***	***Primers***	***Primer's Location***	***Product Length [bp]***
FGF10	**ENSECAG00000014361**	F: GAGATGTCCGCTGGAGAAAG<br>R: TGCTGTTAATGGCTTTGACG	Exon 1 - 2	162
MMP2	**ENSECAG00000000953**	F: TCCCTTTCCTCTTCAACGGC<br>R: CCGTATTTGCCGTCCTTGTC	Exon 4	112
MMP9	**ENSECAG00000013081**	F: CGTGTTTCCCTTCACCTTCG<br>R: GGTCGTAGTTGGCGGTAGT	Exon 6	101
MMP14	**ENSECAG00000008351**	F: CATGATCTTCTTCGCTGAGGG<br>R: GGTGTCGCCTCCAATGTTG	Exon 4	109
MMP15	**ENSECAG00000000196**	F: AGAAGGAGGCCGACATCATG<br>R: CCAGGGAAATAGGCGTGGG	Exon 5	103
MMP17	**ENSECAG00000013201**	F: CACTACGCCCTCAAAGTCTG<br>R: AGGGATAGCGGTCATTGTGG	Exon 4	118
MMP24	**ENSECAG00000024778**	F: TGAGCTGGACACACGGAAA<br>R: TGCCTCTTTCCGGTCACTTT	Exon 4	115
PTGER2	**ENSECAG00000009713**	F: GTCTGCTCCTTGCCTTTCAC<br>R: AAGACCCAGGGGTCGATAAT	Exon 1 -2	122
RECK	**ENSECAG00000010426**	F: GCAGAACGGACGCACTTAC<br>R: GGGCAGGGGTTAGGATTACA	Exon 17	117
TIMP1	**ENSECAG00000014259**	F: CAACCAGACCACCTTACAGC<br>R: ACAAACCAGGTGTCAGGGG	Exon 2 - 3	96
