# Supplementary figures and images for: The Induced Expression of BPV E4 Gene in Equine Adult Dermal Fibroblast Cells as a Potential Model of Skin Sarcoid-like Neoplasia
Source: Int J Mol Sci. 2022 Feb 10;23(4):1970. doi: 10.3390/ijms23041970 (PMC8877312; doi:10.3390/ijms23041970)

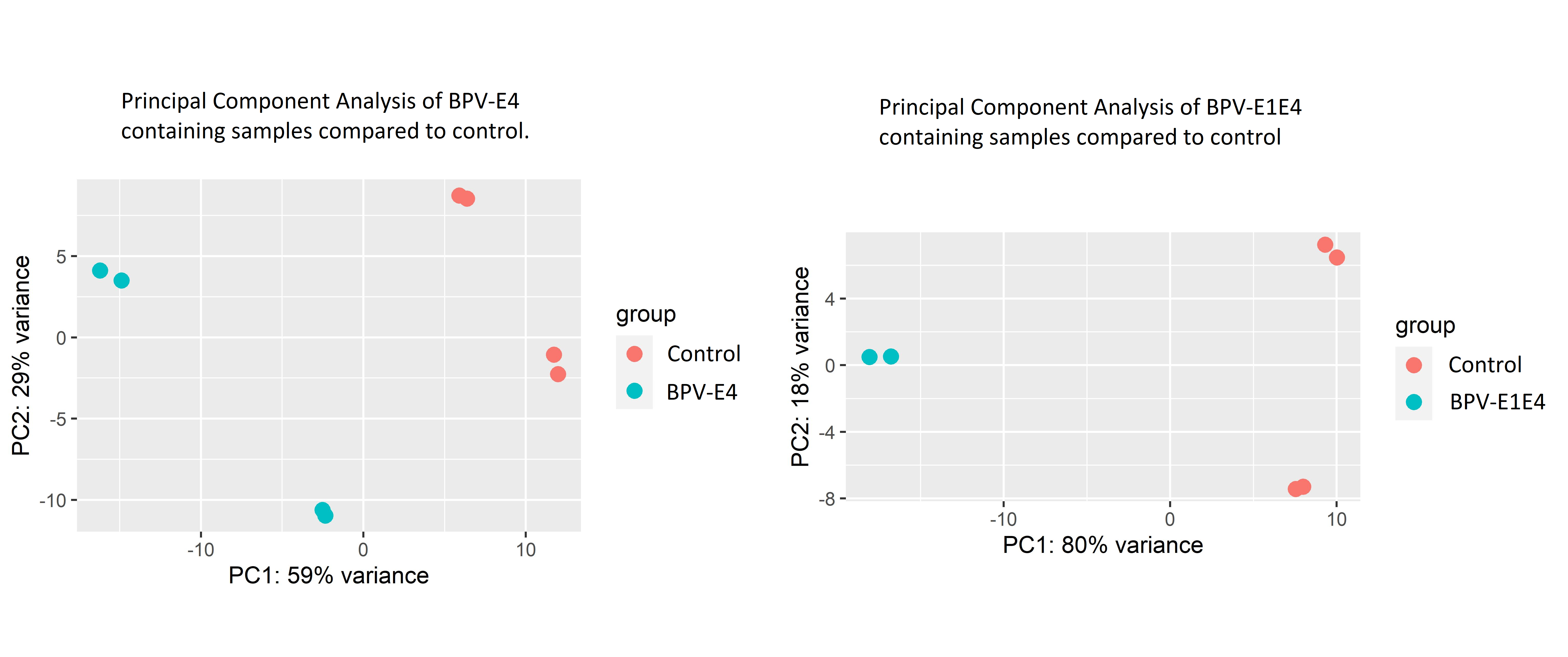

Supplement: Supplementary file 1 [file ijms-23-01970-s001.zip › Supplementary Figure S1 Principal Component Analysis.png]
